# Cocaine in surface waters: a new evidence-based tool to monitor community drug abuse

**DOI:** 10.1186/1476-069X-4-14

**Published:** 2005-08-05

**Authors:** Ettore Zuccato, Chiara Chiabrando, Sara Castiglioni, Davide Calamari, Renzo Bagnati, Silvia Schiarea, Roberto Fanelli

**Affiliations:** 1 Department of Environmental Health Sciences, Mario Negri Institute for Pharmacological Research, Via Eritrea 62, 20157 Milan, Italy; 2 Department of Biotechnology and Molecular Sciences, University of Insubria, Via Dunant 3, 21100 Varese, Italy

## Abstract

**Background:**

Cocaine use seems to be increasing in some urban areas worldwide, but it is not straightforward to determine the real extent of this phenomenon. Trends in drug abuse are currently estimated indirectly, mainly by large-scale social, medical, and crime statistics that may be biased or too generic. We thus tested a more direct approach based on 'field' evidence of cocaine use by the general population.

**Methods:**

Cocaine and its main urinary metabolite (benzoylecgonine, BE) were measured by mass spectrometry in water samples collected from the River Po and urban waste water treatment plants of medium-size Italian cities. Drug concentration, water flow rate, and population at each site were used to estimate local cocaine consumption.

**Results:**

We showed that cocaine and BE are present, and measurable, in surface waters of populated areas. The largest Italian river, the Po, with a five-million people catchment basin, steadily carried the equivalent of about 4 kg cocaine per day. This would imply an average daily use of at least 27 ± 5 doses (100 mg each) for every 1000 young adults, an estimate that greatly exceeds official national figures. Data from waste water treatment plants serving medium-size Italian cities were consistent with this figure.

**Conclusion:**

This paper shows for the first time that an illicit drug, cocaine, is present in the aquatic environment, namely untreated urban waste water and a major river. We used environmental cocaine levels for estimating collective consumption of the drug, an approach with the unique potential ability to monitor local drug abuse trends in real time, while preserving the anonymity of individuals. The method tested here – in principle extendable to other drugs of abuse – might be further refined to become a standardized, objective tool for monitoring drug abuse.

## Background

The use of cocaine, one of the most potent and addictive illicit drugs, appears to be increasing in some countries [1-3]. International drug agencies suggest that this should be closely monitored, in particular among young people in urban areas [3]. The trends and magnitude of drug abuse are currently estimated indirectly from general statistics mainly based on population surveys, consumer interviews, medical records, and crime statistics [3,4]. These general indicators, however, may not realistically estimate the phenomenon at the regional level, where specific socio-economic and cultural patterns can strongly influence drug abuse habits and trends. Moreover, since self-reporting of socially censured behavior is likely to be unreliable, the figures obtained by interviewing known or potential users may be underestimates. New methods are therefore needed not only to provide more realistic estimates of illicit drug consumption, but also to promptly detect changes in abuse trends in local populations. Such methods would therefore help social scientists and the authorities to respond to changing habits with appropriate preventive countermeasures, in "real time".

Several studies, including our own, have reported that therapeutic and veterinary drugs excreted by humans and animals end up in the aquatic environment through the sewage system [5-9]. We collected evidence that environmental levels of largely used therapeutic drugs approximately reflect the total amounts consumed by the local population, as calculated from prescription figures [10-12]. Thus, when factors such as the drug's pharmacokinetics and metabolism and the environmental fate of excretion products are appropriately taken into account, the environmental loads (amounts entering the environment over time) of a drug and/or its major metabolites can become indicators of the drug's consumption by the local population. The idea of possibly using "non-intrusive drug monitoring at sewage treatment facilities" "to determine collective drug usage parameters at the community level" was proposed by Daughton [13] in 2001 but, to our knowledge, has never been implemented.

In the present study we tested whether the above approach could be used to estimate the community consumption figures for a common drug of abuse, namely cocaine. As for therapeutic drugs, in fact, excretion products of cocaine consumed in a given area could in principle be trackable in local waste water (WW) and the receiving surface waters (SW). These environmental compartments can be viewed in fact as a sort of transient "depository" for any sufficiently stable compound excreted by the local population. Thus, finding an excretion product of cocaine in WW and SW could be used to help estimate local consumption. Moreover, if monitored regularly, changing drug concentrations in WW or SW could reflect changes in drug use in real time.

In humans, only a small percentage of a cocaine dose is excreted in urine as the parent drug, while a large amount is excreted as benzoylecgonine (BE) [14,15]. BE is in fact the metabolite often measured in urine to obtain evidence of cocaine use in forensic medicine. Therefore, we searched for and measured both cocaine and BE in aquatic environmental samples, but used concentrations of BE to calculate cocaine consumption more accurately (see Methods).

**Figure 1:**
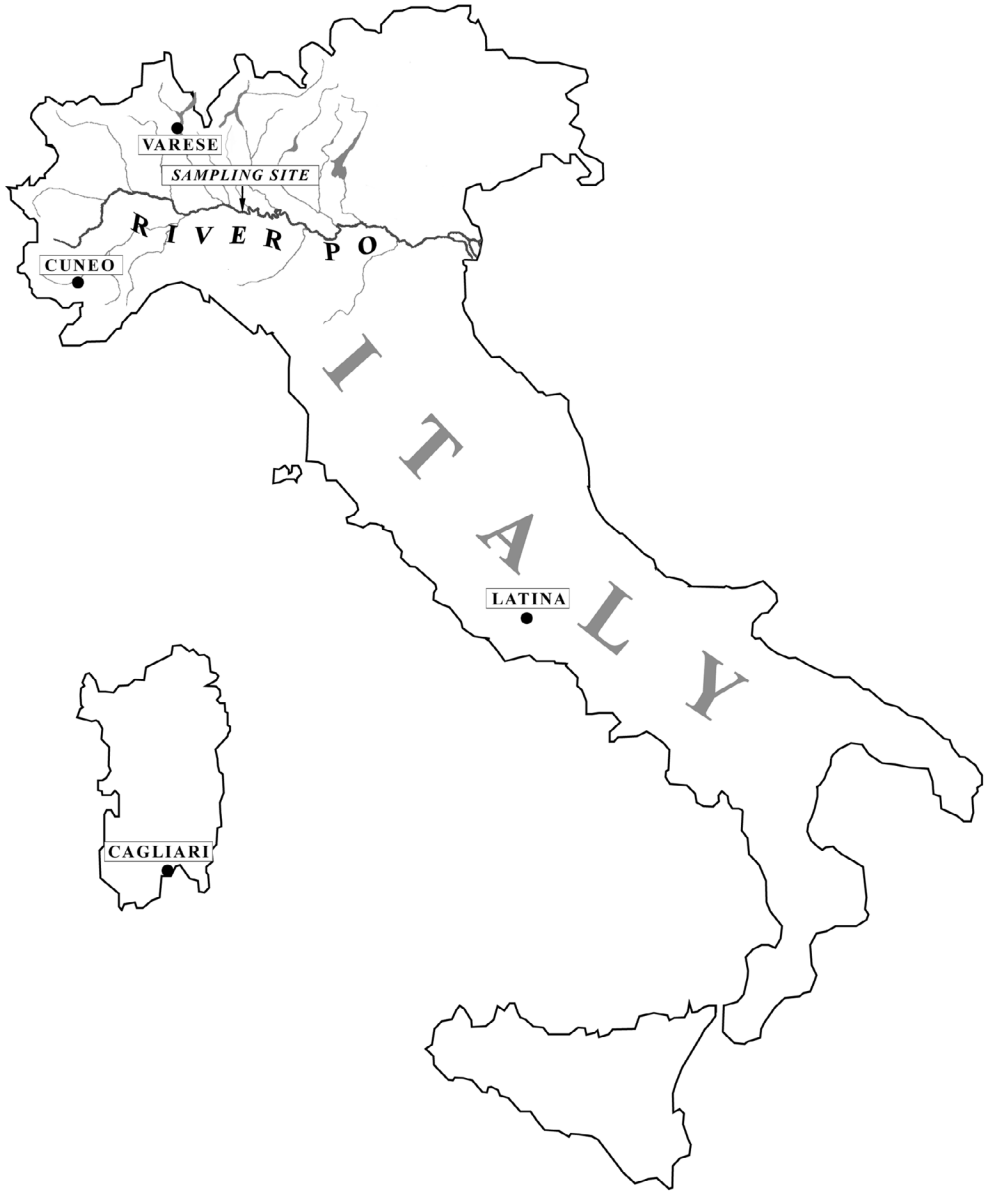
**Sampling sites for cocaine measurement**. Map of Italy showing the River Po basin with the site of sampling, and the locations of the urban waste water treatment plants.

The method used here may obviously – in this first rather unrefined field application – have some intrinsic limitations (discussed below) in the accuracy of collective consumption estimates. Nevertheless, we felt it was worthwhile to test whether or not this evidence-based approach offered a significant improvement over existing indirect methods. After having identified cocaine and BE in the aquatic environment, our main goal was initially to verify how our consumption estimates compared with official figures. We expected our field data on cocaine consumption to give estimates within the range of the official estimates, or perhaps lower, but certainly not higher. In fact, the type of consistent evidence collected from environmental samples and the assumption made for our calculations (see Methods) might possibly lead to under-estimates but hardly over-estimates of the true cocaine consumption. A certain (still unknown) fraction of cocaine excretion products, entering the sewage system from a myriad of scattered inlet points, could in fact be lost and/or degraded before reaching the common sampling site, thus resulting in under-estimates of the true consumption figures. And, again, if we consider that cocaine metabolites sampled from WW and SW cannot reasonably come from sources other than human excretion (apart from sporadic but highly unlikely cases of cocaine disposal into the sewage system or rivers), and that their concentration in flowing waters cannot reflect accumulation, we must once more conclude that we could not have over-estimated true values. With these caveats, we therefore tested this approach on the Italian territory (Figure 1), and compared our findings with official figures for cocaine use in Italy, obtained from surveys of the general population [2].

## Methods

### Chemicals and Materials

The reference standards (99% purity) of cocaine and BE were from MacFarlan-Smith Ltd (Edinburgh, UK), and LGC Promochem s.r.l. (Milan, Italy), respectively. The internal standard (IS), salbutamol-D_3 _(99.1% D) was from CDN Isotopes (Quebec, Canada). Standards were dissolved in methanol at 1 mg/ml and subsequently diluted to 10 ng/μl. Purity of the solutions was checked before each analytical run by HPLC-MS-MS. All solutions were stored at -20°C in the dark. The cartridge used for solid phase extraction was a 3-ml disposable OASIS MCX (60 mg, Waters Corp., Milford, MA).

### Sample collection

Composite water samples (pools of five 500-ml samples collected every 30 min) were collected on four different days from the River Po at Mezzano, Pavia (Figure 1). At this sampling site (average flow rate for the period, 743 m^3^sec^-1^) the basin's population equivalent is 5.4 × 10^6^. The flow rates were kindly provided by the Ufficio Mareografico ed Idrografico del Po. Water samples were also taken from influent WW at four treatment plants (WWTPs) serving medium-size Italian cities (Cagliari, Latina, Cuneo, and Varese; location shown in Figure 1). Flow rates of the WWTPs were 1.0, 0.36, 0.22 and 0.46 m^3^sec^-1^, and population equivalents 270, 140, 45 and 110 × 10^3^, respectively. For each plant, a 24-h, two-liter composite sample was obtained by pooling water collected every 20 min by an automatic sampling device. Water samples were stored at 4°C, and processed within 3 days, to minimize possible degradation.

### Solid phase extraction

Cocaine and BE were measured by adapting our method for pharmaceuticals in river water [10]. Water samples (500 ml) were filtered on a glass micro-fiber filter and spiked with 10 ng of the IS. The pH was then adjusted to 2.0 with 37% HCl. Oasis MCX cartridges were conditioned before use by washing with 6 ml methanol, 3 ml MilliQ water and 3 ml water acidified to pH 2. Samples were then passed through the cartridges under vacuum, at a flow rate of 20 ml/min. Cartridges were vacuum-dried for 5 min and eluted with 2 ml methanol, and 2 ml 2% ammonia solution in methanol. The eluates were pooled and dried under an air stream.

### Liquid chromatographic separation

Before analysis, samples were re-dissolved in 100 μL acetic acid 0.01% in water (pH 3.5), then centrifuged and transferred into glass vials. Aliquots of 10 μl were injected using an auto sampler. The HPLC system consisted of two Series 200 pumps and a Series 200 auto sampler (Perkin-Elmer, Norwalk, CT). A Luna C8 column 50 mm × 2 mm i.d., 3 μm particle size (Phenomenex, Torrance, CA, USA) was used for the chromatographic separation. The elution started with 100% of eluent A (formic acid 0.1% in water, pH 2) followed by a 10-min linear gradient to 100% of eluent B (acetonitrile), 2-min isocratic elution and a 2-min linear gradient to 100% of eluent A, which was maintained for 6 minutes to equilibrate the column. During the analysis the flow rate was 200 μl/min and the column was kept at room temperature.

### Mass spectrometry (MS-MS)

An API 3000 triple quadrupole mass spectrometer (Applied Biosystems – Sciex, Thornhill, Ontario, Canada) was used for quantitative determinations. Analyses were done in ESI positive mode, with a spray voltage of 5.4 kV, orifice skimmer voltages that varied from 30 to 54 V and ring electrode voltages from 180 to 280 V. Data acquisition was performed with multiple reaction monitoring (MRM) of selected fragmentation products of the protonated pseudo-molecular ions (*m/z *290 -> 105 and 290 -> 168 for BE, 304 -> 105 and 304 -> 182 for cocaine, 243 -> 151 and 243 -> 169 for the IS). Five-point calibration curves were generated for each compound by injecting 10 μl of 0.01% acetic acid solutions containing known amounts (0–1 ng/μl) of BE and cocaine, and the IS (0.1 ng/μl). Calibration curves run with each batch of samples showed excellent linearity (r^2 ^> 0.998). Instrumental blanks (standard solution with IS only), showed no traces of interfering compounds. Procedural blanks and recoveries were performed using mineral water. Blanks showed no detectable cocaine and BE. Recoveries were >90% for both compounds. The limits of detection were respectively 0.06 and 0.12 ng/liter for BE and cocaine (calculated as the concentration giving a signal-to-noise ratio of 3 in recovery tests). The identity of cocaine and BE, and the absence of interfering compounds, were further confirmed by MS/MS qualitative analyses performed on a LCQ DecaXP Plus (Thermo Electron, Waltham, MA) ion trap mass spectrometer. In this case, chromatographic conditions were the same previously described, while the mass analysis was made by acquiring ESI-MS and MS/MS spectra, corresponding to the pseudo-molecular ions of BE and COC. The relative amounts of the fragment ions of the substances were in accordance (+/-20%) with those of reference standards.

### Calculations and assumptions

Given that about half a cocaine dose is excreted in urine as BE, and only a small fraction as the unchanged drug, we used the concentrations of BE in WW or SW to estimate the amounts of cocaine consumed locally. Concentrations of cocaine were useful to verify that the BE/cocaine ratio was stable and in the expected range, thus giving confidence about their source being human consumption. If an unlikely accidental or intentional disposal of a significant amount of cocaine were to occur at any of these sites, the normal BE/cocaine ratio (see Results) would be transiently and markedly altered in favor of cocaine. BE loads (g/day) at each sampling site – calculated from the BE concentration in water (ng/liter) and water flow rate (m^3^/sec) – were used to estimate the loads of parent cocaine, multiplying by a factor of 2.33. This takes into account the BE/cocaine molar mass ratio (0.954) and the average molar fraction (45%) of a cocaine dose that is excreted as BE, according to different studies [14,15]. Cocaine loads were then related to the local population equivalents (i.e. the number of people served by a WWTP or living in the river's catchment basin), using data from the Italian 14th General Population and Housing Census (2001) [16]. The estimated consumption (g per day per 1000 people) at each site was referred both to the general population and to young adults (15–34 y), since the latter group reportedly includes almost all consumers [2]. The data were also expressed as the number of doses per day per 1000 people, assuming 100 mg as an average dose [1] (the equivalent of four 25-mg "lines" of cocaine).

## Results

Cocaine and BE were found in all WW and SW samples tested. Concentrations at the various sampling sites are shown in Table 1. As expected, the parent drug levels were much lower than the metabolite, their ratio in WW samples (0.15 ± 0.03, mean ± SD) being in accordance with the known metabolic fate of cocaine in humans. In the River Po, the cocaine/BE ratio was stable over time but lower than expected (0.05 ± 0.02), suggesting a different pattern of degradation and/or partition for cocaine and BE in WWTP and environmental media.

**Table 1:** Levels and loads of cocaine and its metabolite (benzoylecgonine, BE) in the River Po and WWTPs.

	**Levels**^a^		**Loads**
	Cocaine (ng/liter)	BE (ng/liter)	Cocaine equivalents^b ^(g/day)

**River Po**	1.2 ± 0.2^c^	25 ± 5^c^	3800 ± 720^c^
**WWTPs**^d^			
Cagliari	83	640	130
Cuneo	76	420	30
Latina	120	750	33
Varese	42	390	36

^a^Cocaine and BE were analyzed by HPLC-MS/MS

^b^Cocaine loads were estimated from BE concentrations in the waters (see Methods)

^c^Mean ± SD

^d^Waste water treatment plant locations

On four different occasions, at the same sampling site, the River Po was found to steadily carry almost 4 kg of cocaine equivalents per day (Table 1). This suggests a total of about 40,000 doses per day, or about seven doses for every 1000 people living in the river's basin. However, considering only young adults, the estimated use reaches 27 doses per day per 1000 people (Table 2). In agreement with these findings, cocaine loads determined at WWTPs gave drug consumption estimates of about 2–7 doses per 1000 people, or 9–26 doses per day per 1000 young adults (Table 2).

**Table 2:** Local use of cocaine in the River Po basin and medium-size Italian cities, as estimated from BE levels in waters

	**Estimated local cocaine use**			
	
	per 1000 people		per 1000 young adults^a^	
	
	g/day	no. doses^b^/day	g/day	no. doses^b^/day
**River Po**	0.70 ± 0.13^c^	7.0 ± 1.3^c^	2.7 ± 0.5^c^	27 ± 5^c^
**WWTPs**^d^				
Cagliari	0.47	4.7	1.7	17
Cuneo	0.21	2.1	0.9	9
Latina	0.73	7.4	2.6	26
Varese	0.32	3.2	1.4	14
	0.44 ± 0.23^c^	4.4 ± 2.3^c^	1.7 ± 0.7^c^	17 ± 7^c^

^a^15–34 yr old

^b^1 dose = 100 mg

^c^Mean ± SD

^d^Waste water treatment plant locations

## Discussion

The method we have initially tested here with cocaine, as a possible new tool to monitor collective consumption of illicit drugs, gave reproducible estimates from the WWTP, confirmed on a larger scale by the River Po data. However, if this method were to be used in general for continuous monitoring of local drug use, it would be preferable to use repeated sampling in a given, well characterized setting. Sampling WW for drug analysis before it enters a TP would avoid changes in drug concentrations due to removal or degradation that might occur within the TPs. Levels of an illicit drug in a major river of a heavily populated area could still serve to help evaluate consumption on a larger than local scale, but only in those cases where a drug is fairly stable in the aquatic environment.

Clearly, the method implemented here needs to be refined and validated, and adapted for other drugs of abuse before it can become a general tool for monitoring drug abuse. The main aspects to be thoroughly validated involve the chemical and biological stability of the drug's main excretion products and its partition in sewage. Of less concern, in our opinion, is some inaccuracy in estimating consumption that may derive from assumptions related to pharmacokinetics and metabolism. In fact, if consumption is back-calculated from the levels of excreted products using an average drug-to-metabolite fractional conversion factor from multiple thorough studies, the accuracy of the estimate would be only marginally affected by this parameter.

Our data suggest that actual cocaine consumption may be much greater than estimated by current methods. This is a striking result, considering that – as discussed above – the method employed and the assumptions made could only lead to underestimated consumption figures. There is in fact no reasonable mechanism by which cocaine excretion products could accumulate in flowing surface waters, and we found steady concentrations in the River Po over time. Moreover, having chosen in our study to monitor an abundant metabolite in addition to the parent drug, any increase in cocaine levels due to illicit disposal rather than human use would be promptly disclosed by a transient increased cocaine/metabolite ratio.

Official statistics [2] for the year 2001 indicate that in Italy about 1.1% of young adults (15–34 yrs old) admit having used cocaine "at least once in the preceding month", but the actual dosages and frequency of use are not known. Therefore, it is hard to estimate the amount of cocaine that is consumed by the population. If we consider that in the River Po basin there are about 1.4 million young adults, the official figures in this area would translate into at least 15,000 cocaine use events per month. We however found evidence of about 40,000 doses per day, a vastly larger estimate. The economic impact of trafficking such a large amount of cocaine would be staggering. The large amount of cocaine (at least 1500 kilograms) that our findings suggest are consumed per year in the River Po basin would amount, in fact, to about $150 million in street value (based on an average US street value of $100 per gram [17,18]).

The above estimates – obtained from the heavily populated basin of the largest Italian river – were confirmed by similar values found in a completely different setting, i.e. in urban WW of medium-sized cities, chosen in widespread geographical locations to estimate local cocaine consumption on a small scale. The fair correspondence of SW and WW findings, despite the different settings and assumptions, suggests that our approach is reliable, and our estimates realistic. The rather narrow variation of estimated consumption among WWTPs may reflect different local habits, as the urban areas chosen have some socio-cultural differences.

## Conclusion

Surveys of the general population are useful to describe patterns of drug abuse, but they are very expensive, and certainly too lengthy to detect changing trends promptly [4]. Continuous monitoring of illicit drug consumption would be very important for assessing the actual extent of this phenomenon, and detecting changes in trends. A more realistic picture of local use patterns for the most common illicit drugs would also be needed to identify priority problems and plan selective countermeasures. The evidence-based approach first tested here, which is in principle adaptable to other illicit drugs, could be refined and further validated to become a general, rapid method to help estimate drug abuse at the local level. This approach [13], with its unique ability to monitor changing habits in real time, could be helpful to social scientists and authorities for continuously updated appraisal of drug abuse.

## Abbreviations

WW – waste waters

SW – surface water

WWTP – waste water treatment plant

BE – benzoylecgonine

MS – mass spectrometry

## Competing interests

The author(s) declare that they have no competing interests.

## Authors' contributions

EZ designed the study and wrote the paper. CC analyzed the data and wrote the paper. SC collected and analyzed the samples. DC designed the study and analyzed the data. RB developed the analytical method and supervised the analyses. SS collected and analyzed the samples. RF had the original idea, and critically reviewed the results and the manuscript. All authors read and approved the final manuscript.
